# Female, to Nervous and Mental Diseases

**Published:** 1893-03

**Authors:** 


### Selections and Abstracts.

### THE RELATION OF GENITAL IRRITATION, IN THE MALE AND FEMALE, TO NERVOUS AND MENTAL DISEASES.

The above was the title of an interesting paper read by Dr. L. C. Gray, of New York, at the recent meeting of the New York State Medical Society.

A careful review of the history of genital iritation was first given, dating back to the belief of Hippocrates, (which has been the source of the modern doctrines upon the subject,) and this was followed by a discussion, in some detail, of the literature which sprang up after the publication of Stanley's paper, in 1833, as well as the further revival of the question by Sayre, in 1870.

Dr. Gray then went on to say that there was no proof that genital irritation was capable of causing any of the organic diseases of the nervous system, but that it might possibly act as an exciting or aggravating factor of certain lesser nervous diseases. In mental diseases, however, relief of genital irritation has proved, in his opinion, to be a much more valuable method of treatment than it seemed to be in the lesser nervous affections. He called attention, however, to the fact that there were two great classes of mental diseases—namely, the organic insanities and those which are known as the psychoneuroses—and that, so far as was yet known, it was only in the latter that relief of the genital irritation was of any value. He then gave the histories of several classes of insanity in which operations upon the female genitalia had given prompt and permanent relief. He was not, however, willing to believe that all the relief was due to the removal of the genital irritation, because simple etherization had sometimes worked almost as well, and, in one case, had done quite as much as the operation itself would have effected. He furthermore called attention to the fact, that if the operation upon the female genitalia was of any value at all in the relief of mental disease, it should only be done at a proper stage of convalescence. His conclusions were as follows :

1. That there is no proof that genital irritation, in the male or female, can cause nervous or mental disease, except in the predisposed individual.

2. That the proof is not yet absolute that genital irritation can produce nervous or mental disease, even in the predisposed individual.

3. That there is undoubted proof that relief of genital disease, in the male or female, will often relieve certain nervous diseases, such as migraine, hysteria, epilepsy, simple nervousness, and hallucinatory insanity.—*Medical and Surgical Reporter.*

